# Associations between physical activity and motivation, competence, functioning, and apathy in inhabitants with mental illness from a rural municipality: a cross-sectional study

**DOI:** 10.1186/s12888-017-1528-3

**Published:** 2017-11-06

**Authors:** Anders Farholm, Marit Sørensen, Hallgeir Halvari, Torfinn Hynnekleiv

**Affiliations:** 1 Department of Coaching and Psychology, Norwegian School of Sport Sciences, Oslo, Norway; 2 School of Business and Social Sciences, University College of Southeast Norway, Hønefoss, Norway; 3 Innlandet Hospital Trust, Department for Acute Psychiatry and Psychosis, Reinsvoll, Division of Psychiatry, Vestre Toten, Norway; 4 Research & Development Department, Division of Mental Health Services, Akershus University Hospital, Lørenskog, Norway; 5 Institute of Clinical Medicine, University of Oslo, Oslo, Norway

**Keywords:** Physical activity, Motivation, Competence, Mental illness, Apathy, Functioning

## Abstract

**Background:**

There is increasing evidence for physical activity (PA) having a positive impact on physical and mental health as well as illness symptoms in individuals with severe mental illness (SMI). However, individuals with SMI experience several barriers that makes it difficult to take advantage of the benefits associated with PA. One barrier consistently reported to impede PA is motivational issues. Thus, the main aim of the present study was to examine associations between PA and motivation for PA, perceived competence for PA, functioning, apathy, and demographic variables among individuals with SMI. This was conducted within a larger study aiming at including all inhabitants with SMI in one particular small, rural municipality.

**Method:**

A total of 106 participants were recruited to the study. Questionnaire-based interviews conducted by two mental health nurses assessed self-reported PA, motivation and competence for PA, functioning, and apathy. Additionally, 71 participants accepted to wear an accelerometer-equipped wristwatch yielding an objective assessment of PA.

**Results:**

The participants engaged in little PA. However, they did not lack motivation, as over 90% stated that they would like to be more active, and participants across PA level displayed high scores of a motivation reflecting that they valued the benefits of PA. Results showed that higher self-reported PA level was associated with higher levels of integrated regulated motivation and perceived competence for PA while it was unrelated to functioning and apathy. In the subpopulation with objectively measured PA, integrated regulated motivation for PA remained significantly associated with PA level, whereas poor scores on functioning lowered the odds ratio for higher PA level.

**Conclusion:**

The results show that PA specific motivation is associated with PA even when controlling for functioning and apathy. This highlight the importance of facilitating context specific motivation (i.e., motivation for PA) and that health care practitioners should emphasise helping people with SMI develop more intrinsic forms of motivation.

## Background

It is well established that people with severe mental illness (SMI) have increased mortality and morbidity compared with the general population. Their life expectancy is reduced by around 15–20 years, and they display disproportionally high prevalence of somatic diseases [1, 2]. An increased risk of suicide explains part of the reduced life expectancy [3], but the major contributor is physical illness, in particular cardiovascular diseases [4–6]. A large number of factors contribute to the poor physical health of people with SMI [7], including factors related to individual life-style choices, such as smoking, poor diet, excessive drinking, and physical inactivity [8]. Use of physical activity (PA) to improve the health and wellbeing for the general population have long been well acknowledged [9–11]. More recently, there is growing evidence of the beneficial effect of PA on both the physical and mental health also among people with SMI [12–16]. Thus, it is unfortunate that individuals with SMI engage in little PA [17–19]. There can be several reasons for this low PA engagement. Side effects of medicine, illness symptoms, and physical health comorbidities are consistently reported as major barriers for participating in PA [20–22]. Another debilitating factor could be related to motivation. Health care practitioners and patients have reported lack of motivation to be a major obstacle for starting and maintaining PA [23–25]. Hence, there is an increasing interest for examining the motivation for PA among individuals with SMI. However, there is still a great need for improving and refining motivational strategies employed with this population [26–28]. A theoretical framework that have been applied to understand the role of motivation in general [29] and in PA in particular [28] is the Self-Determination Theory (SDT; 30).

In SDT, motivation is proposed to be multidimensional and residing along a continuum between amotivation (indicating a lack of motivation and intention for PA) and intrinsic motivation (performing PA for its own sake due to it being stimulating or enjoyable). Between those extremes, there are four more or less extrinsic motivational regulations. The most extrinsic is external regulation, which means that one is physically active in order to avoid punishment or obtain tangible rewards. The next is introjected regulation, where PA is performed due to internal pressure, such as guilt or contingent self-worth. The two last regulations are more volitionally oriented. In identified regulation the individual personally values the importance of PA, while in integrated regulation PA behaviour is in coherence with personally endorsed values, goals, and needs [31].

Competence is a central concept in several motivational theories e.g., [32, 33]. SDT operates with two concepts of competence, perceived competence and the need for competence. The former refers to perceptions of being able to control important outcomes [34] while the latter refers to a feeling of being effective in producing desired outcomes and exercising one’s capacities [31]. Although the two concepts are to some degree used interchangeably [31], one can say that perceived competence is more concerned with an individual’s evaluation of one’s ability to perform a behaviour. The need for competence is more concerned with whether the individual feels competent, as a result of satisfying feedback from the social context, while performing the behaviour. In that way, perceived competence is similar to self-efficacy as it relates to peoples’ capability to act and perform well [32, 35]. Perceived competence was chosen over need for competence in this study. The reason for this is that assessing participants’ evaluation of their competence of being regularly physically active instead of their perceptions of how competent they felt during PA was more in line with previous studies examining competence or self-efficacy for PA in persons with SMI [36–38].

Competence or self-efficacy for PA and more intrinsic forms of motivation have been associated with more PA engagement among people with SMI [28]. However, to our knowledge there are no studies examining such associations that also take into account the influence of negative symptoms and functioning. It is imperative that this is investigated because poorer functional outcome has been associated with reduced functional exercise capacity and more negative symptoms have been associated with lower PA participation and lower intrinsic forms of motivation for PA [39–41].

In light of the demonstrated benefits of PA and the low PA engagement among individuals with SMI, a better understanding of PA correlates is important for developing improved programs that could increase PA in people with SMI. Thus, the primary aim of this study was to examine associations between PA and motivation for PA, perceived competence for PA, functioning, apathy, and demographic variables among all individuals identified with SMI in a rural municipality. A secondary aim was to examine PA level using both self-report and objective measurement in participants taking part in a study primarily *not* focused on PA.

## Methods

### Design and procedure

The present study is part of a larger study examining the experience of the health care services of patients with SMI. This study aimed to include all inhabitants in one particular rural municipality (~14,800 inhabitants total) having SMI (i.e. severe, long lasting, and recurrent dysfunctional mental problems). Criteria for SMI was primarily operationalised as having at least two hospital stays every year during the last three years, independent of the patients diagnoses. Individuals could also be included if they fulfilled either of the two following conditions; not being able to maintain independent basic living conditions due to confirmed psychiatric disorders, or being followed up regularly by local psychiatric services, but without actual admissions due to refusal or clinical expectations of little or no treatment effect. Participants were excluded if they were not able to give written consent.

A cross-sectional design was applied and data were collected through a questionnaire-based interview. Two clinically experienced and research trained mental health nurses conducted the interviews between May 2013 and April 2015. To ensure inter-rater reliability both mental health nurses conducted pilot interviews, which afterwards were presented, rated and discussed with the principal investigator (T.H.) of the whole project. The principal investigator is an experienced psychiatric clinician who is also a formally certificated research clinical rater with high interrater reliability scores for several of the clinical instruments used. Although no formal reliability analyses were performed, the pilot ratings were considered highly satisfactory as they were identical or near-identical on all the crucial items, both internally between the mental health nurses and between the mental health nurses and the principal investigator.

The interviews lasted approximately two hours and were carried out at a place convenient for the participant, often in their own residence. If participants got tired during the interview, it would be divided into two parts. After completion of the interview, participants were debriefed. If requested by the participant, or the interviewer considered it appropriate, participants were followed-up by competent health-care personnel. The study was approved by the Regional Committee for Medical and Health Research Ethics (approval number 2013/154), and participants gave written informed consent.

### Participants

The mapping of all inhabitants with SMI in the municipality required a comprehensive identification process. Potential participants were identified through examining electronic lists of individuals utilising public mental health service in the municipality and in the relevant mental health hospital and outpatient clinics. The catchment area of the mental health hospital included the whole municipality. The project included all key clinicians in the municipality and this provided a very good general overview of the psychiatric status in the municipality. This thorough examination located 208 individuals satisfying the inclusion criteria. Of those, 61 declined to participate, typical reasons were lack of energy, interest, health problems, as well as no reason at all. Additionally, some initially accepted the invitation, but did not meet for the scheduled interview. Furthermore, 15 had moved to another municipality when contacted by the interviewers and were thus not eligible. Finally, the interviewers were not able to establish contact with 26 potential participants. This resulted in a total of 106 participants being interviewed in the study (i.e., 0.7% of the general population in the municipality).

### Measures

#### Physical activity

PA was assessed with both self-report and objective measurements as recommended by Lindamer and colleagues [42]. Participants were also asked if they had any wish to increase their PA level. Self-reported vigorous, moderate, and walking PA during the last 7 days was measured with International Physical Activity Questionnaire short version IPAQ: [43]. At the very end of the interview, the participant was asked whether he or she would be willing to wear an accelerometer-equipped wristwatch (Polar Active, Polar Electro Oy, Kempele, Finland) to measure PA objectively. Polar Active measurement technology has been validated by measuring energy expenditure using indirect calorimetry [44]. Participants who accepted wore the Polar Active monitor in the consecutive week after the interview. Activity was measured in five different intensities: very easy (e.g. watching TV), easy (e.g. slow walking), moderate (e.g. gymnastics), vigorous (e.g. dancing), and vigorous + (e.g. fast running). Only the three latter intensities are considered to be PA [45]. Daily wear time was calculated as the sum of all five intensity zones. The days where activity monitors were distributed and collected were not included in the analyses. To be included for analysis, participants had to have >600 min of wear time on at least 4 days.

Participants were grouped according to their PA level. We followed established guidelines for self-reported PA that categorize participants into groups of low, moderate and high activity level [46]. We added a fourth category called “no activity” for those participants reporting no activity during the last week. Furthermore, it was decided to collapse the moderate and high activity level categories because few participants ended in the high activity level group (*n* = 13). For objectively measured PA participants were divided into two groups: 1) <30 min of PA, and 2) ≥30 min of PA. This distinction was based on how typical guidelines for PA recommend doing at least 150 min of moderate PA each week (e.g. 30 min on most days) [47].

#### Motivation for physical activity

Motivation for PA was measured according to SDT using the Treatment Self-Regulation Questionnaire [48]. Participants were presented the question "The reason I am physically active is…", and then responded to statements assessing integrated regulation (three items, e.g. "because to be physically active have become an incorporated practice for me"), identified regulation (four items, e.g. "because I want to take responsibility for my own health"), introjected regulation (two items, e.g. "because I would not feel well if I'm not physically active"), external regulation (e.g. three items, "because I want other people to see that I can do it"), and amotivation (three items, e.g. "I really don't know why I should do it"). Responses were made on a 7-point scale ranging from 1 (*not true*) to 7 (*very true*). The mean score (range 1–7) was used for each motivational regulation and high scores indicate high levels of that specific motivational regulation.

#### Perceived competence for being in regular physical activity

Perceived competence for being in regular PA was assessed according to SDT using the Perceived Competence Scale [49]. The scale contains four items (e.g. "I manage to be in regular physical activity right now") and responses were made on a 7-point Likert scale ranging from 1 (*not true*) to 7 (*very true*). The mean score (range 1–7) was used and high scores reflects high levels of perceived competence for being in regular PA.

#### Health and social functioning

The Health of the Nation Outcome Scale (HoNOS) was developed by British College of Psychiatrist specifically to assess health and social functioning for people with SMI [50]. The scale consists of 12 clinician rated items covering four domains: 1) behaviour (e.g. self-injury and problem drinking), 2) function / impairment (e.g. cognitive problems and physical illness), 3) symptoms (e.g. problems with depressed mood or delusions), and 4) social functioning (e.g. problems with relationships or activities of daily living). Each item is scored from 0 (no problem) to 4 (severe/very severe), yielding a total score ranging from 0 to 48 where low scores indicate better functioning. HoNOS is a not infrequent used mental health outcome measure in clinical practice as well as in Norwegian mental health research projects. For descriptive purposes, participants were stratified into groups of severity of impairment in function based on their HoNOS scores on items 1–10, except item 5. The four groups were ‘very severe’ (two or more item’s score ≥ 3), ‘moderately severe’ (one item’s score ≥ 3), ‘clinical’ (one or more item’s with a score of 2), and ‘subclinical’ (score 1 or less on all items). This procedure is proposed by Lelliott [51] and later refined by Parabiaghi, Kortrijk and Mulder [52].

#### Apathy in life in general

Apathy was assessed based on Marin’s [53] conceptualisation of apathy as "diminished motivation and goal directed behaviour, not attributed to diminished level of consciousness, general cognitive impairment, or emotional distress". In the present study we used a 12-item Short Version of the Apathy Evaluation Scale SH-AES: [54]. Example items are "It is important for me to get things done during the day" and "Someone has to tell me what to do every day (reversed)". Participants indicate how well this characterised them on a scale ranging from 1 (*very much*) to 4 (*not at all*). This yielded a total score ranging from 12 to 48 where high scores indicate high levels of apathy. The prevalence of clinical apathy was also estimated. A score of 27 in the SH-AES was used as cut-off value for indicating clinical apathy [55].

### Data analysis

All data were entered in EpiData Entry (EpiData, Odense, Denmark) and checked for accuracy by a repeated check. All statistical analyses were conducted using IBM SPSS statistics for Windows, Version 21 (IBM Corp., Armonk, New York, USA). To assess potential differences in variables according to PA level we performed Kruskal-Wallis test or one-way analysis of variance (_ANOVA_), as appropriate. If a main effect was present, this was followed by post-hoc procedure including Mann-Whitney *U*-test or independent *t*-test. For categorical variables chi-square tests were conducted. Statistical significance was set at *p* < 0.05 and Bonferroni corrections were applied in post-hoc procedure. The association between PA level and the other variables were further examined using multivariate logistic regression models. Following the procedure of Jiménez et al. [56] only variables associated with PA level with a significance of *p* < 0.10 in the univariate comparisons were entered as independent variables. ‘No activity’ (self-report) and ‘<30 min’ (objectively measured) were chosen as reference groups. Mean and median with belonging standard deviation and percentiles were used to describe self-report and objective measured PA among participants.

## Results

### Participants

The 106 participants in the study had a mean age of 45.7 (SD 11.9), 65 (61.3%) were female, and 45 (42.5%) were in a relationship. Approximately ^3^/_4_ of the individuals were raised either in the same municipality (*n* = 55 [51.9%]) or county (*n* = 22 [20.8%]) as the present study recruited participants from. The remaining participants were brought up either elsewhere in Norway (*n* = 24 [22.6%]) or abroad (*n* = 5 [4.7%]). The mean number of hospitalisations and years since first contact with mental health service were 7.3 (SD 13.0) and 13.1 (SD 6.1), respectively. The distribution of PA level groups, primary ICD-10 diagnosis, and HoNOS severity category are shown in Table 1. Fifty-one (49.0%) of the participants rated themselves to be apathetic according to the predefined cut-off value of 27.

**Table 1:** Distribution of PA level group, primary mental illness diagnosis, and HoNOS severity categories

	Frequency	
Self-report PA level group		
No activity	21	(21.0%)
Low active	43	(43.0%)
Moderately active	12	(12.0%)
High active	13	(13.0%)
Objective PA level group		
Less than 30 min/day	25	(45.5%)
30 or more min/day	30	(54.5%)
ICD-10 primary diagnosis		
F 10–19	21	(19.8%)
F 20–29	11	(10.4%)
F 30, 32–39	34	(32.1%)
F 31	11	(10.4%)
F 40–49	20	(18.9%)
F 60–69	5	(4.7%)
F 70–79	1	(0.9%)
F 90–98	3	(2.8%)
HoNOS severity category		
Very severe	17	(16.7%)
Moderately severe	58	(56.9%)
Clinical	11	(10.8%)
Subclinical	16	(15.7%)

### Preliminary analyses

Continuous data were tested for normality using the Kolmogorov-Smirnov test and by visual inspection of Q-Q plots and data distribution in histograms. These analyses indicated that only age and years of contact with mental health service had normally distribution of data. Internal consistency was measured with Cronbach’s *α*. The variables integrated regulation (*α* = 0.97), identified regulation (*α* = 0.79), external regulation (*α* = 0.65), perceived competence (*α* = 0.90), and apathy (*α* = 0.80) had acceptable *α* values. Introjected regulation (*α* = 0.30) and amotivation (*α* = 0.20) had *α* values well below what is typically regarded as acceptable [57] and they were removed from further analyses.

Missing data in self-reported PA, the three motivational regulations, perceived competence, functioning, and apathy were less than 4%. To increase statistical power we computed the average for the motivational regulations, perceived competence, and apathy scales if a participant had completed 67% of the items in the respective measure. An advantage of inserting mean values is that a participant’s score remains unchanged, but such practice can decrease the variance in the data [58]. It was deemed improper to impute mean values in HoNOS because each item measure different aspects of functioning that not necessarily have a transferable value between them. Likewise, in the self-reported PA measurement it is recommended to remove participants with missing data from analysis [46].

Seventy-one participants accepted to wear the accelerometer. Of those, 13 did not use the accelerometer enough to calculate a meaningful score of PA and three did not return it. We examined if there were any differences in all variables between participants with and without valid objective measurements of PA. The only significant difference was that participants using the accelerometer self-reported more PA minutes than those not using it (48.2 ± 55.6 versus 29.8 ± 47.4; *U* = 861, *z* = −1.99, *p* = .046).

### Primary univariate analysis

#### Comparison between three levels of self-reported physical activity

As described in Table 2, integrated regulation yielded a significant main effect across self-reported PA level while external and identified regulation did not. Post-hoc analyses showed that there were significant differences between all three levels of PA with higher scores of integrated regulation associated with higher levels of PA. The same pattern as for integrated motivation was evident for perceived competence for PA, with one exception. After correcting for multiple tests the difference between ‘no physical activity’ and ‘low physical activity’ became *non*-significant. There were no differences in functioning, apathy, or sociodemographic variables between the three levels of PA. However, for apathy the Jonckheere-Terpstra test revealed a significant trend (*J* = 1241.5; *p* = 0.020) towards more active participants reporting less apathy even though the main effect was non-significant.

**Table 2:** Univariate analyses of all study variables according to degree of self-report PA level

	No PA (A) *n* = 21		Low PA (B) *n* = 43		Mod/high PA (C) *n* = 36		χ^2^ / *F* / *H*	*p*	*p*		
	Mean (SD)		Mean (SD)		Mean (SD)				A vs. B	A vs. C	B vs. C
Motivational regulations for PA											
Integrated regulation	1.58	(1.12)	3.50	(2.48)	5.40	(2.20)	30.023	**<0.001**	**0.003**	**<0.001**	**<0.001**
Identified regulation	4.88	(2.00)	5.75	(1.44)	5.88	(1.39)	4.824	0.090			
External regulation	1.39	(0.85)	1.85	(1.29)	1.50	(1.04)	3.983	0.136			
Perceived competence for PA	4.10	(2.28)	5.46	(1.78)	6.46	1.17	18.790	**<0.001**	0.022^b^	**<0.001**	**0.004**
Functioning	7.85	(5.10)	7.02	(3.73)	6.42	(3.86)	0.976	0.614			
Apathy	28.52	(7.71)	26.05	(8.42)	23.51	(7.38)	5.385	0.068			
Age^a^	46.8	(13.7)	45.1	(11.7)	45.9	(10.9)	0.149	0.862			
Gender (female [%])	47.6%		60.5%		69.5%		2.665	0.264			
Marital status (relationship [%])	52.4%		39.5%		38.9%		1.179	0.555			
Body mass index $\left(\frac{m^2}{kg}\right)$	26.69	(5.11)	29.64	(5.13)	29.68	(6.87)	3.434	0.180			
Years of contact^a^	14.1	(5.5)	13.2	(5.1)	14.4	(7.1)	0.386	0.681			
Number of hospitalisations	3.62	(3.57)	7.60	(11.82)	9.42	(17.65)	1.851	0.396			
Accelerometer data (yes[%])		38.1%		48.8%		63.9%	3.838	0.147			

^a^Normally distributed variables. Bold types indicate significant test statistic. ^b^ *not* significant after Bonferroni correction. *PA* physical activity

#### Comparison between <30 min and ≥30 min of objectively measured physical activity

As presented in Table 3, the more active participants in the subgroup with valid objective measurement of PA reported significantly higher levels of integrated regulation, identified regulation and perceived competence. There was no difference in external regulation, but participants in the most active group were rated to have significantly better functioning and self-reported significantly lower level of apathy in comparison to participants in the less active group. There were no differences in demographic variables.

**Table 3:** Univariate analyses of all study variables according to degree of objective measured PA level

	< 30 min PA *n* = 25 Mean (SD)		≥ 30 min PA *n* = 30 Mean (SD)		χ^2^ / *U* / *t*	*p*
Motivational regulations for PA						
Integrated regulation	2.52	(1.91)	5.38	(2.34)	133.5	**<0.001**
Identified regulation	5.01	(1.86)	6.02	(1.57)	200.0	**0.011**
External regulation	1.79	(1.41)	1.52	(1.03)	306.0	0.535
Perceived competence for PA	4.20	(2.13)	6.37	(1.11)	135.5	**<0.001**
Functioning	9.86	(3.62)	4.93	(2.89)	80.0	**<0.001**
Apathy	28.89	(6.26)	22.83	(8.03)	205.0	**0.004**
Age^a^	46.2	(10.3)	44.7	(11.3)	0.497	0.621
Gender (female [%])	64.0%		73.3%		0.556	0.456
Marital status (relationship [%])	36.0%		40.0%		0.092	0.761
Body mass index $\left(\frac{m^2}{kg}\right)$	30.35	(7.87)	28.38	(5.19)	317.5	0.435
Years of contact^a^	13.4	(6.0)	15.5	(6.9)	1.198	0.236
Number of hospitalisations	7.68	(18.79)	7.80	(9.40)	292.5	0.161

^a^Normally distributed variables. Bold types indicate significant test statistic

### Primary multivariate analysis

#### Multinomial logistic regression model predicting self-reported physical activity level

The multinomial logistic regression model with ‘no activity’ as reference group showed that higher level of integrated regulation and perceived competence were significantly associated with higher PA level even when controlling for the other variables. The significant odds ratios were larger when predicting ‘moderate/high physical activity’ level compared to ‘low physical activity’ level (see Table 4). This model accounted for 46.4% of the variance in self-reported PA [χ^2^ [10] = 47.61, *p* < .001, Nagelkerke pseudo *R* ^2^ = 0.464].

**Table 4:** Multivariate logistic regression of factors associated with PA level

	Self-reported PA						Objective measured PA		
	Low level PA			High/moderate level PA			≥30 min of PA		
	Wald	*p*	OR (95%CI)	Wald	*p*	OR (95%CI)	Wald	*p*	OR (95%CI)
Integrated regulation for PA	5.059	0.025	**2.03** (1.10–3.78)	9.847	0.002	**2.80** (1.47–5.31)	4.014	0.045	**1.63** (1.01–2.62)
Identified regulation for PA	1.756	0.185	1.34 (0.87–2.06)	0.360	0.548	1.17 (0.71–1.93)	0.035	0.853	0.94 (0.48–1.84)
Perceived competence for PA	5.623	0.018	**1.62** (1.09–2.41)	7.792	0.005	**2.06** (1.24–3.41)	1.792	0.181	1.77 (0.77–4.07)
Functioning	1.189	0.275	1.11 (0.92–1.32)	1.490	0.222	1.14 (0.92–1.41)	7.913	0.005	**0.38** (0.20–0.75)
Apathy	0.240	0.624	1.03 (0.92–1.16)	0.201	0.654	1.03 (0.90–1.17)	1.206	0.272	1.11 (0.92–1.34)

Bold types indicate significant OR. ‘no physical activity’ is reference group for self-reported PA and ‘<30 min of PA’ is reference group for objective measured PA

#### Binary logistic regression model predicting objectively measured physical activity

The binary logistic regression model with ‘<30 min of physical activity’ as reference group showed that higher level of integrated regulation was significantly associated with higher level of objective PA. In this analysis, also lower level of functioning significantly lowered the odds ratio for having higher level of PA (see Table 4). This model accounted for 76.8% of the variance in objectively measured PA [χ^2^ [5] = 41.26, *p* < .001, Nagelkerke pseudo *R* ^2^ = 0.768].

### Physical activity

As seen in Table 1, nearly two thirds of the participants were categorised into either the ‘no activity’ or ‘low active’ group according to their self-reported PA. In the subgroup with objective PA measurement approximately half of the participants engaged in 30 min or more PA each day. Participants reported on average very little vigorous and moderate intensity PA (see Table 5). Additionally, 65 (63.7%) participants self-reported that they did not do any 10 min bouts of vigorous or moderate PA during a typical week. According to the objective PA measurements, 40 (72.7%) participants did on average less than 10 min of moderate and vigorous PA each day. Finally, 94 (90.4%) of the participants reported that they would like to increase their level of PA.

**Table 5:** Minutes of self-report and objective measured PA

	*n*	Mean	(SD)	Median	(25th – 75th)
Self-reported PA					
Total min/day	95	39.3	(52.3)	20	(4–56)
Vigorous intensity	106	5.5	(15.6)	0	(0–0)
Moderate intensity	102	7.4	(20.4)	0	(0–1)
Walking	96	29.0	(40.7)	9	(0–40)
Objective reported PA					
Total min/day	55	44.9	(34.2)	34	(22–65)
Vigorous intensity	55	1.9	(3.4)	1	(0–2)
Moderate intensity	55	7.5	(9.7)	5	(2–8)
Low intensity	55	35.6	(24.3)	27	(18–53)

### Secondary analyses

Even though there were no differences in any variables, except self-reported PA, between those with and without objective measurements of PA the two groups had clearly different patterns of associations (see Table 6). The associations between study variables were stronger for participants with objective data on PA compared to those without. Especially the associations between self-reported PA and functioning and apathy were different between the two groups. This could indicate a moderating effect of accepting to wear the accelerometer on the association between self-report PA and functioning and apathy. This was tested following the procedure of Cohen, Cohen, West, and Aiken [59]. These analyses yielded significant interaction effects. More specifically, in the group who accepted to wear the accelerometer participants with less apathy and better functioning reported more self-reported PA in comparison to those with more apathy and worse functioning. In comparison, level of functioning and apathy did not differ with respect to level of self-reported PA in participants *not* wearing the accelerometer.

**Table 6:** Spearman correlation between study variables

	1.	2.	3.	4.	5.	6.	7.	8.	9.
1. PA self-report (categories)	1	–	.58***	.18	.03	.33*	.17	.01	.90***
2. PA objective (categories)	.30*	1	–	–	–	–	–	–	–
3. Integrated motivation for PA	.54***	.53***	1	.45**	.01	.25	−.09	−.24	.64***
4. Identified motivation for PA	.21	.36**	.36**	1	−.01	.13	−.21	−.21	.14
5. External motivation for PA	−.06	−.09	−.20	.23	1	−.32*	.07	.31*	.06
6. Perceived competence for PA	.61***	.56***	.54***	.27	−.38**	1	−.17	−.27	.38*
7. Functioning	−.37**	−.65***	−.39**	−.28	.06	−.41**	1	.41**	.09
8. Apathy	−.49***	−.39**	−.46***	−.10	.09	−.57***	.62***	1	−.02
9. PA self-report (continuous)	.91***	.31*	.55***	.23	−.04	.53***	−.44**	−.49***	1
10. PA objective (continuous)	.29*	.86***	.37**	.24	−.06	.53***	−.59***	−.42***	.32*

Note: Below the diagonal participants *with* objective PA measurement; above the diagonal participants *without* objective PA measurement; PA variables labeled continuous are PA measured by total minutes of PA; * *p* < .05; ** *p* < .01; *** *p* < .001

## Discussion

To the best of our knowledge, this is a unique sample with regards to examining both PA level and motivation for PA among individuals with SMI. This is the first study combining a very comprehensive recruitment process, in which all potential participants in one municipality were actively approached, with the fact that participants were not biased to accept or decline participation based on their interest for PA because the main aim of the present study was *not* related to PA. Moreover, this is the first study examining PA and motivation and perceived competence for PA that also includes assessment of apathy and functioning.

The main finding from this study is how higher levels of integrated regulated motivation and perceived competence for PA were associated with higher levels of PA even when controlling for functioning and apathy. This is important because it shows that targeting motivation and competence for context specific behaviour (i.e., PA) should be emphasised in treatment. This finding of the importance of integrated regulated motivation and competence for PA is in line with research from the general population [60, 61].

The present results also showed that participants, independent of their PA level, scored very low on externally regulated and relatively high on identified regulated motivation or PA. This suggests that motivation based on rewards and punishment (i.e., external regulation) only plays a distal role in why individuals with SMI engage in PA. Furthermore, it shows that participants were aware of, and valued the benefits of PA, but alone this type of motivation (i.e., identified regulation) did not seem to be enough to transfer intentions into action. This interpretation is in line with participants scoring highest on the statement "I am physically active because I personally believe it is the best thing for my health" (i.e., identified regulation) and the lowest on the statement "I am physically active because I want others to approve of me" (i.e., external regulation).

Functioning and apathy were not associated with self-reported PA. This finding stand in contrast to previous studies showing more negative symptoms and poorer functioning to be related to lower level of PA [39, 40]. However, both functioning and apathy were negatively associated with PA in the subgroup with objectively measured PA. Functioning was also associated with objectively measured PA even when controlling for the other study variables. There were no differences in demographic variables across PA level.

A possible explanation for the equivocal results regarding functioning and apathy can be that the design of the study allowed us to recruit two subpopulations, one with ‘more interest’ and one with ‘less interest’ for PA. We therefore used ‘wearing the accelerometer’ as a behavioural measure of interest in PA to differentiate between the two groups. This method of differentiating is based on the assumption that participants with more interest in PA would be inclined to take on the extra “work load” of wearing the accelerometer for a week. Additionally, they self-reported more PA than those not wearing the accelerometer. Examining the two subpopulations yielded an interesting result. First, there were no differences in any variables, except self-reported PA, between the two groups, but they had obvious different patterns of associations. Functioning and apathy were associated with PA, integrated regulation for PA, and perceived competence for PA in the subgroup with ‘more interest in PA’ while they were not in the group with ‘less interest in PA’. These differences in associations can explain the equivocal results regarding functioning and apathy. To summarise from a clinical point of view, having better or worse functioning and more or less apathy did not influence whether participants were interested in PA, but among those interested in PA, better functioning and less apathy was associated with higher levels of PA.

Participants engaged in little PA and the large majority did not do any moderate or vigorous intensity PA. The same PA pattern was reflected in both type of measurements and the correlation between them (in total minutes of PA) is of similar magnitude as in a study validating the self-report questionnaire in outpatients with schizophrenia [62]. The low PA level is not surprising based on previous research [17–19], and it underscores the importance of increasing the knowledge of barriers and facilitators associated with PA in this population.

An advantage of using a theoretical framework when examining motivation for PA is that evidence suggest that health promotion interventions based on social and behavioural science theories are more effective than those lacking a theoretical base [63]. One particular benefit of applying SDT is that the multidimensional view on motivation allows one to examine how different qualities of motivation are associated with PA. In the present study, participants in general displayed high levels of identified regulation and over 90% reported that they would like to increase their PA level. However, it was only integrated regulation that was associated with higher levels of PA. This indicate that participants *were* motivated for PA, but for the majority of participants their type or quality of motivation (e.g. identified regulation) was not sufficient to start engaging in PA. Thus, participants may need help from family, friends, or mental health care practitioners to develop a more integrated style of motivation.

### Implications

There are several worthwhile reasons for promoting PA. There are the well established benefits of PA to the health and wellbeing for the general population and the growing evidence for PA to be a useful part of treatment for mental illness [9–16]. Moreover, there are studies showing that PA can be an arena for establishing social relationships, obtaining mastery experiences, and contributing to a sense of meaning, purpose, and identity as an active person [64–66]. However, for health-care practitioners working with facilitating motivation for PA among individuals with SMI it is important to be sensitive towards circumstances related to mental illness. For instance, patients can be well aware of the positive benefits of PA and also express interest and intention to engage in more PA, but they may lack the energy or inner drive to be able to execute their intentions. Health care practitioners being too persuasive towards promoting PA could therefore evoke feelings of guilt and inadequacy if the patient for any reason is not able to engage in PA. With that in mind SDT is a suitable framework for facilitating motivation as it emphasize the autonomy of the patient, taking the perspective of the patient and creating an empathic relationship with the patient [30].

There is ample knowledge about how health practitioners can create an environment that facilitate change towards more intrinsic types of motivation among patients [31]. In short, SDT proposes that satisfaction of the three psychological needs of autonomy, competence, and relatedness will facilitate more intrinsic forms of motivation [30]. There are several strategies mental health practitioners can employ to support this process and the following are just a selected sample. Autonomy support can be given through involving patients in the decision-making process, providing rationales for decisions made by practitioners, and by giving relevant choices. An important aspect when supporting the competence need is to adapt the PA context in a way that ensures patients have the opportunity to experience mastery, which is particularly important when introducing new activities. Helping with realistic goal-setting, providing positive feed-back, and suggesting coping strategies for overcoming barriers are other possibilities to support the competence need. Finally, the relatedness need can be supported through creating a warm and welcoming atmosphere, acknowledging patients’ perspective, feelings, and values, and organising the activity so that patients can develop and strengthen attachment with others [67–69].

### Limitations

This study is not without limitations. The cross-sectional design of the study does not make it possible to infer causality, only associations. There is a great need for intervention studies with the main aim of influencing motivation for PA in people with SMI [26, 28]. As all cross-sectional studies this study is prone to common method bias [70]. However, we have tried to minimise this bias by using both self-report and objective measurement of PA and clinician and participant evaluation with regards to functioning and apathy. Finally, information about previous experience with PA could have added valuable insight.

## Conclusion

With the acknowledged positive benefits PA have on mental and physical health in people with SMI it is imperative to develop the knowledge of factors that promote and impede PA engagement in this population. This study adds to that body of knowledge by showing that PA specific motivation is associated with PA even when controlling for functioning and apathy in this sample recruited from one municipality.

## Abbreviations

PA: Physical activity
SDT: Self-determination theory
SMI: Severe mental illness
